# Sexual Enhancement Products for Sale Online: Raising Awareness of the Psychoactive Effects of Yohimbine, Maca, Horny Goat Weed, and *Ginkgo biloba*

**DOI:** 10.1155/2014/841798

**Published:** 2014-06-15

**Authors:** Ornella Corazza, Giovanni Martinotti, Rita Santacroce, Eleonora Chillemi, Massimo Di Giannantonio, Fabrizio Schifano, Selim Cellek

**Affiliations:** ^1^School of Life and Medical Sciences, University of Hertfordshire, College Lane, Hatfield AL10 9AB, UK; ^2^Department of Neuroscience and Imaging, University “G. d'Annunzio”, 66100 Chieti, Italy; ^3^Cranfield University, Bedfordshire MK43 0AL, UK

## Abstract

*Introduction*. The use of unlicensed food and herbal supplements to enhance sexual functions is drastically increasing. This phenomenon, combined with the availability of these products over the Internet, represents a challenge from a clinical and a public health perspective. *Methods*. A comprehensive multilingual assessment of websites, drug fora, and other online resources was carried out between February and July 2013 with exploratory qualitative searches including 203 websites. Additional searches were conducted using the Global Public Health Intelligence Network (GPHIN). Once the active constitutes of the products were identified, a comprehensive literature search was carried out using PsycInfo and PubMed. *Results*. The most common sexual enhancement products available on the Internet were identified. Their active ingredients included yohimbine, maca, horny goat weed and *Ginkgo biloba.* These four substances were reported with the occurrence of adverse events and the induction of psychological symptoms, such as mood changes, anxiety, and hallucinations as well as addictive behaviours. *Conclusions*. Uncontrolled availability of sexual enhancement products that contain potentially harmful substances is a major public health concern. The possible impact on population health, particularly among subjects with psychiatric disorders, usually at risk for sexual dysfunction, may be significant. This new trend needs to be extensively studied and monitored.

## 1. Introduction

The use of food and herbal supplements to enhance erectile function as well as sexual arousal and desire has become increasingly popular in the recent years [1, 2]. These products are advertised on the Internet as “*natural*” and “*safer*” alternatives to pharmaceutical products, such as gold standard for treatment of erectile dysfunction and the phosphodiesterase type 5 (PDE5) inhibitors (e.g., sildenafil, tadalafil, and vardenafil). However, they often contain potent active constituents [3–5], which remain undeclared on the packaging [6, 7]. The effects of the aforementioned products, thus, remain unknown until reports on harms expose the problem and accurate clinical evaluation takes place.

Since herbal supplements are plant products, they are generally perceived and hence marketed as natural and, therefore, “safe” [8]. However, several case reports of side effects to herbs and herbal products have been reported [8, 9], such as on- and off-target effects from biologically active ingredients, side effects caused by contaminants, and herb-drug interactions. It is difficult to determine the true frequency of side effects from herbs and herbal products, since surveillance systems are much less extensive than those in place for pharmaceutical drugs. It is estimated that less than 1% of side effects or adverse events for herbal products are recorded with the current surveillance systems [10].

Some of the side effects and adverse events due to ingestion of herbs and herbal products are related to their action on central nervous system and can present as mood alterations, anxiety, mania, depression, hallucinations, and addictive behaviour; one example of such herbal product is ephedra, which was banned in the USA in 2004 [11].

However, psychological safety profiles of sexual enhancement products have not been characterised. This review, therefore, aimed at identifying the sexual enhancement products advertised and sold on the Internet while collecting information about their characteristics (i.e., brand names, costs, ingredients, availability, declared advantages, and disadvantages). We then focused on four substances, which are common in these products: yohimbine, maca, horny goat weed, and* Ginkgo biloba*, as these were reported to induce psychological side effects. We also described, less in detail, other components of sexual enhancer products that may have both somatic and psychological side effects.

## 2. Methods

A multilingual qualitative assessment of a range of websites, drug fora, and other online resources (i.e., e-commerce, e-newsgroups, chat rooms, videos, e-newsletters, and bulletin boards) was carried out between February 2012 and May 2013. Exploratory qualitative searches of 203 websites in English and Italian took place using brand names through Google search engine. Of these, 106 were considered to be relevant for the study and as such were monitored on a regular basis, that is daily (*n* = 21), weekly (*n* = 32), or monthly (*n* = 53), depending on their relevance. The remaining 97 websites were considered not to bear any interest for the present study and thus were no longer monitored. Further specific searches in the database provided by The Global Public Health Intelligence Network (GPHIN) also took place. This is a secure Internet-based early warning system that gathers preliminary reports of public health significance by monitoring global media sources near “real-time,” 24 hours a day, 7 days a week basis. GPHIN is operated by the Public Health Agency of Canada and monitors news sources and websites across the globe in 9 languages (e.g., English, French, Farsi, Portuguese, Arabic, Russian, Spanish, and Chinese simplified/traditional) [12]. While a series of algorithms were used and adjusted to capture relevant information, analysis of relevant data since 2003 was also carried out manually by a multidisciplinary and multilingual team of analysts. Finally, once the active constituents of these products were identified, a comprehensive literature search was carried out using PsycInfo and Pubmed databases. Permission for the study was granted by the School of Pharmacy Ethics Committee, Hatfield, UK (15 December, 2010; PHAEC/10-42).

## 3. Results

During the website assessment stage where 136 websites were monitored for 6 months, we have identified 15 sexual enhancement products advertised and sold on the Internet. The characteristics of these products are summarized in Table 1. Yohimbe, ginseng,* Ginkgo biloba*, maca, horny goat weed, and L-arginine were claimed to be the most common ingredients in those products. Among all the substances identified in these products, we have found psychological safety concerns for only four: yohimbine, maca, horny goat weed, and* Ginkgo biloba*. Biological, pharmacological, and safety profiles of these four substances are presented below.

### 3.1. Yohimbine

#### 3.1.1. Source and Preparations

Yohimbine is a natural tryptamine alkaloid, which can be extracted from the bark of a variety of plants mostly of African and Asian origin such as* Pausinystalia yohimbe *(also known as yohimbe tree) and the root of* Rauvolfia serpentina*. Yohimbe bark extract is traditionally used in Africa as an aphrodisiac: it has to be underlined, though, that tree bark does contain a large number of alkaloids, and yohimbine level in this natural source may be low. Yohimbine concentration has been suggested to increase in older branches [13]. A 1995 study by Betz et al. on dietary supplements containing Yohimbe extracts has shown that the yohimbine content may range from < 0.1 to 489 ppm compared with 7089 ppm in the authentic material, which has been attributed to the intense dilution of final product [14]. Currently, yohimbine-containing products are advertised on the Internet not only for treating erectile dysfunction and enhancing sexual performance, but also as a fast weight-loss and body-building supplement [15–17]. Brand names include “The Millennium Male Horse Power Capsule” [18], Syntrax [15], and Xytomax (see Table 1).

#### 3.1.2. Mechanism of Action

Yohimbine has high affinity to human alpha-2 adrenoceptors, moderate affinity to alpha-1 adrenoceptors, and low affinity to some of the serotonin and dopamine receptors in the central and peripheral nervous systems [19]. Alpha-2 adrenoceptors mediate erection-inhibiting impulses in the central nervous system. Yohimbine is generally believed to enhance central sexual impulse by blocking the alpha-2 adrenoceptors in the locus coeruleus in the brain [20]. In the periphery, yohimbine has been suggested to inhibit alpha-1 and alpha-2 adrenoceptors as well as enhancing the release of nitric oxide (NO) from cavernosal endothelial cells [21, 22]. In conclusion, the mechanism of action of yohimbine in enhancing sexual function is currently unclear.

#### 3.1.3. Pharmacokinetics

Yohimbine hydrochloride is rapidly absorbed and the maximum plasma concentration is generally achieved in less than one hour after oral administration [23, 24]. The mean bioavailability is low and is subject to very high variation from one individual to another [25]. The plasma concentration of yohimbine does not appear to correlate with the dose of the compound administered [26].

#### 3.1.4. Efficacy

A meta-analysis in 1998 of seven clinical trials with yohimbine has shown a superiority of the compound over placebo in treatment of erectile dysfunction [27]. Yohimbine has also been reported to be effective in treatment of orgasmic disorders such as delayed ejaculation [28]. There have been no clinical studies testing Yohimbe bark. The Clinical Guidelines Panel on Erectile Dysfunction of the American Urological Association (AUA) has concluded that the data available on Yohimbine do not allow it to be recommended as standard treatment in erectile dysfunction particularly not in organic aetiology [29]. International Society for Sexual Medicine (ISSM) Standards Committee has recently stated “*if yohimbine has any potential indications for use in ED management, it would be among nonorganic ED; apart from ED, yohimbine has shown a limited efficacy in the treatment of premature ejaculation*” [21].

#### 3.1.5. Safety

The side effects of yohimbine include high blood pressure, increased heart rate, manic reactions, bronchospasm, palpitations, insomnia, anxiety, irritability, shivering, sweating, nausea, flushing, and headaches which all can be attributed to its central adrenergic activity [26, 30].

#### 3.1.6. Case Reports Concerning Safety of Yohimbine

Adverse events mostly due to self-administration of yohimbine have been reported since 1980s. In 1993, Sandler and Aronson have described the development of progressive renal failure, cutaneous eruption, and a lupus-like syndrome in a 42-year-old Afro-American man following yohimbine use [31]. Myers has reported a case of refractory priapism in a 42-year-old HIV infected and depressed man who self-administered a product containing yohimbine to treat his erectile dysfunction and was subsequently admitted to emergency department with a 20-hour lasting erection which required insertion of a proximal corpus cavernosum to spongiosum shunt (Quackles shunt) [32]. According to California Poison Control System, 238 cases of adverse reactions after yohimbine consumption have been identified within a 7-year period (2000–2006): most commonly reported adverse events have been gastrointestinal distress, tachycardia, anxiety, agitation, and hypertension [33]. US National Institute of Health also warns consumers about taking yohimbine supplements if they suffer from schizophrenia, anxiety, depression, or posttraumatic stress disorder [34]. Additional situation of emergency has been reported in Amman where the public corporation of the food and the medicine warned the citizens about the use of some unlicensed sexual stimulants, such as yohimbine, for its inclusion of poisonous substance strychnine that caused convulsions, hallucinations, and heart and kidney failure [35]. More recently, Yohimbine was detected in a product labelled as OxyELITE Pro and sold online as slimming pills. This caused the hospitalisation of two female patients aged 34 and 21 in Hong Kong for acute hepatitis symptoms. As a result, the Department of Health opened an investigation and appealed to members of the public not to buy or consume the product [30].

#### 3.1.7. Psychological Safety

As mentioned above, anxiety and agitation are among the most common side effects of yohimbine or yohimbine-containing products [33]. US National Institute of Health warns consumers about taking yohimbine or Yohimbe supplements with some of the antidepressants and antipsychosis drugs: “*People should not combine Yohimbe with monoamine oxidase (MAO) inhibitors as effects may be additive. Yohimbe should be used with caution when taken with medicines for high blood pressure, tricyclic antidepressants, or phenothiazines (a group of medicines used mostly for mental health conditions such as schizophrenia). People with kidney problems and people with psychiatric conditions should not use Yohimbe*” [34]. In 1985, Linden et al. have reported a case of a 16-year-old girl who experienced an acute dissociative reaction accompanied by weakness, paraesthesia, incoordination, anxiety, headache, and chest pain after the ingestion of an aphrodisiac called “*yo-yo,*” which has been later identified as yohimbine [36]. Acute neurotoxic effects related to the ingestion of yohimbine-containing products have been reported by Giampreti et al., who has reported the case of a 37-year-old bodybuilder presented with malaise, vomiting, loss of consciousness, and repeated seizures after ingestion of 5 g of yohimbine during a competition [37].

### 3.2. Maca

#### 3.2.1. Source and Preparations

Maca* (Lepidium meyenii)* is a Peruvian plant, which belongs to the Brassicaceae family. It grows in central Andes at more than 4000 m altitude; it is constituted of a flat overground portion and underground hypocotyl and roots. Hypocotyl colour allows the distinction of three different varieties: white, yellow, and black maca. Naturally dried hypocotyls have been used for centuries by native Andean populations as aphrodisiac, energizer, and enhancers of fertility and sexual function [38]. With the advent of Internet, maca has become a common ingredient of sexual enhancer products available worldwide.

#### 3.2.2. Active Ingredients and Mechanism of Action

Maca extracts have been shown to contain benzyl glucosinolates and polyphenols, (1R,3S)-1-methyl-1,2,3,4-tetrahydro-*β*-carboline-3-carboxylic acid (MTCA), and p-methoxybenzyl isothiocyanate among many other chemicals [39]. The mechanism of action of maca in altering sexual behaviour is unknown.

#### 3.2.3. Pharmacokinetics

No information could be found related to the pharmacokinetics properties of Maca.

#### 3.2.4. Efficacy

Zheng et al. reported increased sexual behaviour in male rats and mice and decreased latency to erection in testes-removed rats following oral administration of lipid extracts of maca roots [40]. Cicero et al. demonstrated that sexual behavioural activity as well as locomotor activity was increased in rats treated with pulverised maca root orally [41]. In a further study, the same group has demonstrated that the hexanic extract of maca had more effect on sexual behaviour of rats than the methanolic and chloroformic extracts [42]. Lentz et al. measured ejaculatory and mounting behaviour and postejaculatory intervals in rats treated with maca orally for 30 days. They found that maca had only a small effect on sexual behaviour and locomotor activity but no effect on anxiety [43].

The studies with maca on human subjects were reviewed in 2010 [44]. Four randomised clinical trials met the authors' inclusion criteria: two trials suggested a significant positive effect of maca on sexual dysfunction or sexual desire in healthy postmenopausal women [45] or healthy adult men [46], while the other trial failed to show any effects on healthy cyclists [47]. A further trial assessed the effects of maca in patients with erectile dysfunction using the International Index of Erectile Function Questionnaire and showed significant effects [48]. Lee et al. have reviewed the literature to determine maca's possible effects on menopausal symptoms and confirmed the inadequacy of the present data to clearly state efficacy and safety [49]. An overview of systematic reviews on various complementary medicine products for sexual dysfunction in elderly patients has found that the evidence for maca was insufficient [50].

#### 3.2.5. Safety

Piacente et al. (2002) have shown the existence of 1R,3S-1-methyl-1,2,3,4-tetrahydro-*β*-carboline-3-carboxylic acid (MTCA) in the extracts of maca. MTCA has been suggested to be an inhibitor of the monoamine oxidase (MAO) enzyme and is a comutagenic or a precursor to mutagenic compounds [51]. Because of these potential mutagenic properties of MTCA, the French Agency for Sanitary Security warned consumers of powdered maca root about possible health risks from its ingestion [52]. These findings have been disputed by others based on the assumption that MTCA is deactivated during the boiling process of the maca roots [53]. MTCA-like compounds have also been suggested to be associated with craving behaviour, which is common in addictions [51]. Side effects subjectively reported by fora users were altered menstrual cycles, moodiness, cramps, gastritis, and insomnia (see Table 2).

### 3.3. Horny Goat Weed

#### 3.3.1. Source and Preparations

*Epimedium* is a genus of plants of the Berberidaceae family, including more than 50 species. Among the most frequently used in sexual enhancer products are* Epimedium grandiflorum* and* Epimedium sagittatum*.* Epimedium grandiflorum* is commonly known as horny goat weed named after the legendary discovery of its aphrodisiac properties, attributed to a Chinese goat herder who noticed an increased sexual activity in his herd after they ate the plant's leaves [54], and sold online with various names including ViaXtreme, which also contains undeclared sildenafil [17].

#### 3.3.2. Active Ingredients and Mechanism of Action

The active ingredient of horny goat weed has been suggested to be icariin, a flavonol glycoside [55]. Icariin and its derivatives have been suggested to increase NO synthesis in the penis [56], inhibit PDE5 in cavernosal smooth muscle [57–59], have positive neurotrophic effect on nitrergic nerves [56], enhance smooth muscle proliferation [60], and decrease advanced glycation endproduct formation [60] and mimic endogenous androgens [61]. Icariin has also been suggested to have anti-inflammatory, antiosteoporotic, and neuroprotective properties [62–64].

#### 3.3.3. Pharmacokinetics

Icariin has been shown to have low oral bioavailability, poor absorption, and short plasma half-life [65]. Because of these unfavourable pharmacokinetics profiles, liposome encapsulated formulations of icariin are being developed [66].

#### 3.3.4. Efficacy

Although icariin has been shown to be efficacious in treatment of erectile dysfunction in aged [67], diabetic [68], and castrated [69] animal models, it has not been tested in any randomised clinical trial.

#### 3.3.5. Safety

No long-term toxicity study has been conducted using horny goat weed (or icariin) in any animal species. Partin and Pushkin have presented a case of a 66-year-old man with a history of cardiovascular disease who, after two weeks of consumption of herbal sexual enhancer containing horny goat weed, was admitted to hospital with a new-onset tachyarrhythmia and hypomanic symptoms (sexual and verbal inappropriate behaviour, irritable mood, and hyperverbal speech). The patient needed to undergo an electric cardioversion and to be treated with a therapy based on olanzapine and lorazepam [70]. Metz et al. reported a case of a painful vasculitic rash in a 77-year-old man, who had started a treatment with* Ginkgo biloba* and horny goat weed tablets four days before. The man improved after three days as an inpatient, with the suspension of the herbal supplement [71].

#### 3.3.6. Psychological Safety

As mentioned above, one case has been reported with hypomanic symptoms combined with tachyarrhythmia [70]. During the online monitoring of discussion fora, several cases of aggressive behaviour and irritability were noted (see Table 2).

### 3.4. *Ginkgo biloba*

#### 3.4.1. Source and Preparations

*Ginkgo biloba* is a living fossil tree, belonging to Ginkgoaceae family. Typically grown in China, Ginkgo plants are tall, normally reaching 20–35 m of height, with fan-shaped leaves. The extracts obtained from Ginkgo leaves contain flavonoid glycosides (e.g., myricetin and quercetin) and terpenoids (e.g., ginkgolides and bilobalides) and have been used for centuries in traditional Chinese medicine, particularly for memory enhancement and blood flow improvement.

#### 3.4.2. Active Ingredients and Mechanism of Action

The most commonly used* Ginkgo biloba* extract is EGb761, which contains flavonoid glycosides, mainly composed of kaempferol, quercetin glucorhamnoside esters, and terpenes of ginkgolides and bilobalides. These ingredients have been suggested to have the ability to inhibit MAO enzyme and uptake of certain neurotransmitters such as noradrenaline and serotonin in the central nervous system [72]. A recent meta-analysis of 28 clinical studies with Ginkgo has found no significant effect on cognitive function [73]. Ginkgo extracts have been suggested to improve sexual function by improving the blood flow to the brain as well as to the genital organs. These extracts have also been suggested to increase NO bioavailability, which may have positive impact on sexual function [74].

#### 3.4.3. Pharmacokinetics

Extracts of Ginkgo are a mixture of substances with a wide variety of physical and chemical properties and activities. A recent review on the pharmacokinetic properties of these substances concluded that the available pharmacokinetic data are rare and more studies need to be conducted [75]. It is generally accepted that Ginkgo extract is well absorbed in humans, rats, and rabbits after oral administration [75].

#### 3.4.4. Efficacy

Ginkgo extracts have been tested as a treatment for antidepressant-induced sexual dysfunction, which is a common side effect in depressive disorder therapy, especially when using selective serotonin reuptake inhibitors (SSRIs). Despite the initial positive effect on sexual function based on various case reports [76], two randomised placebo controlled trials have found no significant benefit on sexual function with Ginkgo extracts [77, 78]. Although Ginkgo extracts have been suggested for the treatment of female sexual arousal disorders [79], no placebo controlled randomised trial has been reported.

#### 3.4.5. Safety

A recent long-term study by National Toxicology Program has demonstrated that oral administration of Ginkgo extract caused cancer of thyroid gland and liver, atrophy of the olfactory epithelium, nephropathy, and mononuclear cell leukaemia in rats and mice [80]. One of the major ingredients, quercetin, is a known mutagen [81]. In reviews of the efficacy and safety of Ginkgo extracts in human subjects, no serious side effects were reported in clinical trials and side effects were similar to those seen in the placebo groups [82]. Ginkgo extract has been suggested to affect platelet aggregation, which may not only be useful in prevention of pathological events as thrombosis, but may also cause spontaneous bleeding in healthy individuals or patients treated with anticoagulants such as warfarin and aspirin [83]. There have been case reports of subdural hematoma or cerebral haemorrhage with headache, confusion, blurred vision, nausea, and vomiting after Ginkgo administration [84]. Some Ginkgo extracts may contain 4′-O-methylpyridoxine, which is a neurotoxin also known as “ginkgotoxin.” Ginkgotoxin poisoning is characterised by epileptic convulsions, vomiting, unconsciousness, and irritability, which may be fatal if not promptly treated [85].

#### 3.4.6. Psychological Safety

Ginkgo may interact with other medications particularly those acting on the central nervous system. It may decrease (alprazolam, citalopram, and diazepam) or increase (clozapine, methadone, olanzapine, and fluvoxamine) effects and side effects of such drugs; it may also induce hypomanic symptoms when taken together with St. John's wort, fluoxetine, and melatonin [86]. Anxiety was the most common psychological effect reported by Ginkgo users at Internet discussion fora (see Table 2).

### 3.5. Other Substances

Velvet antler has been used in traditional Chinese medicine for centuries, mainly as an energizer and to treat joint pain, cardiovascular diseases, and impotence. The name refers to the velvety skin covering growing antlers of male elk, deer, and moose, which can be removed with no harm for the animal. In western countries, elks and deer are anesthetized with xylazine or lidocaine before having velvet removed; this practice had determined an ethical controversy among veterinarians, to find balance between animal welfare and food safety (not being known drugs' withdrawal time for velvet) [87]. The most relevant issue currently identified for elk or deer velvet antler consumption is related to an infectious neurodegenerative prion disease known as chronic wasting disease (CWS). The core symptoms of this encephalopathy are notable weight loss and behavioural abnormalities (e.g., progressive hyperexcitability) [88]. Though currently interspecies transmission has not been yet demonstrated, the presence of CWS prions in animal muscle, fat, glands, and antler velvet may expose humans to infection by handling or consumption of diseased cervids products [89, 90]. No psychoactive properties have been yet demonstrated for velvet antler.

*Turnera diffusa,* also known as “damiana,” is a small shrub common in Mexico, Central and South America, and the Caribbean.* Turnera diffusa* leaf is traditionally thought to be stimulant, aphrodisiac, tonic, diuretic, laxative, and useful to treat menstrual and pregnancy disorders [91]. Aphrodisiac and psychoactive properties have been ascribed for centuries to damiana. Its leaves are usually boiled to make an infusion with the aim of intensifying sexual sensations; moreover, when smoked, damiana leaves may produce similar effects to marijuana [92]. It has recently been found in products advertised on the Internet to increase sensuality, especially among women [16], as legal and safer alternative to* Cannabis* and sold as “mystical incenses” [93, 94]. These also often contain synthetic cannabinoids such as JWH-18, JWH-73, and HU210 that may cause severe intoxication and death [95, 96].

*Nymphaea caerulea* is a lotus flower belonging to Nymphaeaceae family. It is also known as “Blue Water Lily” or “Egyptian Lotus” and it is sold as a powder named “Blue Lotus.” The flower has been used by ancient Egyptians not only to enhance sex drive and to improve sexual performance, but also to stimulate blood flow and as an antiageing treatment. It has also recreational potential when added to wine or smoked, due to its psychoactive properties [97]. The flower may indeed induce both narcotic and euphoric effects and, if taken in large amounts, may cause slightly hallucinogenic symptoms [98].

*Ptychopetalum olacoides* (*Muira puama*), also called “potency wood,” grows primarily in Brazil. Popular belief claims that it can improve sexual function especially in old men [7]. This herb may be found in plant markets or in herbal formulations, usually sold to increase physical, mental, and/or sexual performance. Few clinical studies have so far examined the prosexual effects of* Muira puama*. Waynberg reported in 1994 that 60% of men with low libido have reported increased sexual desire and 50% of men with poor erection have reported improved erectile function following* Muira puama* administration [99]. Another study has shown that* Muira puama* improved sexual desire, sexual fantasies, and the ability to achieve orgasm in 65% of women with prior sexual dysfunction [100].

#### 3.5.1. Synthetic Substances

Among synthetic substances used as sexual enhancers, well-known examples include MDPV (methylenedioxypyrovalerone), known as “Ivory Wave,” 2C-B, and mephedrone (4-methylmethcathinone), also called with the nickname of “Miaow Miaow.” The aforementioned substances have stimulating and aphrodisiac properties similar to amphetamines and MDMA (“Ecstasy”). Because of their partially synthetic and partially natural composition, some sexual enhancer substances are defined "mixed." Lust is a stimulant made up of* Sida cordifolia* extract. This plant contains L-ephedrine, which is a natural amphetamine acting on both central and peripheral nervous systems. The effects of L-ephedrine are mainly linked to an expansion of sexual perception [101]. Another component of Lust is saw palmetto (*Serenoa repens*,* Serenoa serrulata*), another natural aphrodisiac; the plant's berries are used to increase muscular power and libido. The last Lust component is L-arginine, an amino acid believed to have positive effects on impotence and infertility [102].

## 4. Discussion and Conclusions

The exponential increase in the use of unlicensed sexual enhancing products, often characterized by the undeclared presence of psychoactive agents, represents a serious challenge both from clinical and public health point of view, despite their large and uncontrolled distribution on the Internet, including e-commerce websites (Ebay, Amazon). It has been estimated that approximately six million men in the EU purchase these products from the Internet [103]. Possible reasons behind such a risky behaviour include (a) the embarrassment to speak with a doctor, (b) the thought that buying them online would be the fastest and cheapest way to solve the problem, (c) curiosity, (d) peer-pressure, and (e) the increase of sexual confidence and performance [5].

In a performance-based society, the e-commerce of food and herbal supplements sold for sexual enhancing purposes is becoming a highly profitable market as a result of low-cost manufacturing capacity in countries such as China and India as well as the globalization of free trade and the Internet as a platform for rapid distribution [94, 103, 104]. In this context, unscrupulous manufacturers/retailers are exploiting the common belief that “natural” products are healthier alternatives to pharmaceutical treatments and infiltrate undeclared harmful psychoactive substances in their products [5]. This represents a growing public health threat, which is putting the population at risk. The frequent occurrence of side effects and the potential induction of symptoms of psychiatric interest, such as mood elevation, anxiety, hallucinations, and other psychotic symptoms, are issues that need to be clarified and constantly monitored at the international level [105, 106]. The possible impact on subjects with psychiatric disorders, usually at risk for sexual disjunctions and therefore possible consumers, may be devastating.

Sexual function is closely related to the function of the central nervous system; it is therefore not surprising that psychological and psychiatric disorders are closely associated with sexual dysfunction [107]. For example, depression can be a cause or result of erectile dysfunction. Some of the antidepressants can also cause sexual dysfunction. It is therefore essential that patients with sexual dysfunction either of psychogenic or organic aetiology should avoid taking such herbal products which may have adverse effects on their mental state. Similarly, patients taking centrally acting medications such as antidepressants or antipsychotics should avoid using such products which may cause unwanted herb-drug interactions and hence side effects, one of which may be sexual dysfunction.

In addition, there is still a distinct paucity of evidence-based and up-to-date information available for medical professionals on the effects and risks associated with the consumption of these products. In this sense, the use of unstructured, free, and near real-time sources of information such as Internet news and online reports is essential to rapidly acquire and share knowledge and understanding of the rapidly changing drug market and be better prepared to face this new challenge globally.

A limitation of this study was that only publicly available websites and similar sources were monitored. In order to improve the coverage of the study, not only the websites but also more private ways of communication (including newsgroups, chat rooms, mailing lists, social networking, e-newsletters, bulletin boards, and videos) were monitored. Because some of the discussion fora have restricted access to non-registered members, a comprehensive quantitative analysis could not always be fully performed. A further limitation may be that the present findings do rely mostly on what is reported by users. We did not have any possibility to ascertain whether the substance the online alleged drug users were taking was only a sexual stimulant or a combination of various products.

Considering the fact that only less than 1% of the side effects are reported for herbal products, it is concerning to find the variety of psychological symptoms disclosed by the users in online discussion fora, which may only be a fraction of the tip of an iceberg. In conclusion, the four substances, yohimbine, maca, horny goat weed, and* Ginkgo biloba,* commonly found in sexual enhancement products carry potential safety risks particularly in psychological context. The users, physicians, and regulators should be aware of these risks.

## Figures and Tables

**Table 1:** Characteristics of sexual enhancer products sold online.

Brand name	Ingredients	Cost	Availability	Effects/declared advantages from anecdotal online reports	Side effects/disadvantages from anecdotal online reports
Endowmax http://www.endowmax.com/	*Epimedium grandiflorum* (horny goat weed); maca; damiana; L-arginine; *Tribulus terrestris*; gamma-aminobutyric acid; jujube dates extracts; Muira puama (potency woods); Catuaba bark; *Xanthoparmelia scabrosa*; *Cnidium monnieri*; Tongkat Ali (*Eurycoma longifolia*)	59.95 $ per box; six-month supply for 239.95 $	Online suppliers (orders can be placed by fax and phone); Ebay; Amazon (currently out of stock).	Powerful and natural; no unpleasant side effects; customers testimonials; money back guarantee; it is clearly stated the product does not contain Yohimbe.	Up to 8 weeks for initial benefits; expensive.

Xytomax http://www.xytomax.com/	Niacinamide (vitamin B3); zinc oxide; *Epimedium sagittatum* (standardized to 10% icariin); maca root; Guarana extract (standardized to 20% caffeine); Korean ginseng root; L-arginine; Muira puama; Longjack extract; *Avena sativa*; Yohimbe bark extract (2% yohimbine); *Ginkgo biloba* extract (24% flavonglycodises & 6% terpene lactones); saw palmetto (berry); *Xanthoparmelia Scabrosa*; *Cnidium monnieri* extract; GABA; damiana (leaf)	69.96 $ per box; six-month supply for 309.95 $	Online suppliers (orders can be placed by fax and phone); Ebay.	Natural; working from the very first dose; customers testimonials; money back guarantee.	It is not to be used in case of thyroid, kidney, or heart diseases or high blood pressure; more expensive than other products.

Xanogen http://www.xanogen.com/	*Epimedium grandiflorum* (horny goat weed); maca; Yohimbe; L-arginine; *Tribulus terrestris*; gamma-aminobutyric acid; Muira puama (potency woods); Catuaba bark; *Xanthoparmelia scabrosa*; *Cnidium Monnieri *	89.95 $ per box; six-month supply for 419.95 $	Online suppliers (orders can be placed by fax and phone); Ebay; Amazon (currently out of stock).	100% natural products; money back guarantee; no negative side effects; permanent effects on corpora cavernosa.	It could cause stomach upset if not taken with food; it does not provide immediate erections but stronger ones.

Virility EX http://www.virilityex.it/	Yohimbe; maca; Elk Antler Velvet; Eleuthero; Longjack; Muira puama (potency woods); Catuaba bark; oat straws.	29.89 € per box; six-month supply for 89.74 €	Online suppliers (orders can be placed by fax and phone); Ebay; Amazon (currently unavailable).	Stated as: “No side effects; 100% natural; money back guarantee.”	Some users claim no results with this product; just a couple of customer testimonials were found on official websites with no FAQ sections.

Enzyte http://www.enzyte.com/	Korean red ginseng; *Gingko biloba*; grape seed extract; horny goat weed; Muira puama; niacin; zinc.	39.95 $ per box; six-month supply for 179 $	Online suppliers; Ebay; Amazon.	Premium natural ingredients; no gluten, no preservatives, no ephedra, no caffeine, no Yohimbe.	Reported hives, facial flushing, insomnia, and internal bleeding; some users claim no results with this product.

Extagen http://www.extagen.com/	Not clearly listed on website, just “herbs and phytonutrients.”	59 $ per box; six-month supply for 198 $; twelve-month supply for 288 $	Online suppliers (orders can be placed by phone, and mail too); Ebay; Amazon.	Natural, vegetarian and hypoallergenic; money back guarantee; it is clearly stated the product does not contain Yohimbe; customers testimonials.	Contraindicated in case of high blood pressure or other medical conditions.

Extenze http://www.extenze.net	L-Arginine; Eleutherococcus; Yohimbe bark extract; nettle; ginseng; folate; micronized DHEA; Muira puama; pregnenolone; zinc; black pepper; *Piper longum*; ginger. *Cnidium monnieri*; *Astragalus*: *Xanthoparmelia Scabrosa*; boron; GABA; velvet deer antler; horny goat weed; damiana; pumpkin; licorice extract; Ho Shou Wu extract.	59.95 $ per box; six-month supply for 305.95 $; ten-month supply for 499.95 $	Online suppliers (orders can be placed by fax, phone, and mail too); Ebay.	Stated as: “all natural; assures penis enlargement in addition to increasing sexual stamina; customers testimonials; money back guarantee.”	Legal controversies; reported increased heart rates, agitation, and nervousness.

Maxidus http://www.maxidus-pill.com/	*Eurycoma longifolia*; *Flos catharmi*; Rizoma *Curcumae longae*; *Gingko biloba*; Herba Epimedii; Herba Cistanches; *Astragalus membranaceus*; *Momordica charantia* L (Ku Gua).	39.95 $ per box; six-month supply for 399.75 $; twelve-month supply for 479.40 $	Online suppliers (orders can be placed by fax, phone, and mail too); Ebay.	Stated as: “works in 10–30 minutes and lasts for up to 4 days; money back guarantee; 100% natural; it can be taken by people with heart or prostate problems, hypertension, diabetes; it can be used by women too.”	Slight headache reported; expensive; clinical tests carried out are shown for only one ingredient (*Eurycoma longifolia*).

Orexis http://www.orexis-pills.co.uk	*Tribulus terrestris*; Epimedium sagittatum; Muira puama; *Panax ginseng*; Catuaba bark extract; damiana; Yohimbe extract.	29.16 *£* per box; six-month supply for 135.00 *£*; twelve-month supply for 262.50 *£*	Online suppliers (orders can be placed by fax, phone, and mail too); Ebay.	Stated as: “all natural; works within 45 minutes; customers testimonials; money back guarantee.”	Legal issues; interaction with antidepressants, diuretics, and high blood pressure medications.

Pro Solution Pills http://www.prosolutionpills.com/	Solidilin; Korean ginseng; Butea superba; *Momordica*; apigenin; Amla; Arjuna; *Cordyceps*; zinc oxide: reishi mushroom; *Curculigo*; drilizen; bladderwrack.	59.95 $ per box; six-month supply for 289.95 $; twelve-month supply for 399.95 $	Online suppliers (orders can be placed by fax, phone, and mail); Ebay; Amazon.	Stated as “all natural; no side effects; multiple orgasms per sexual encounter; customers testimonials; money back guarantee.”	Reported nausea, hypertension; increased heart rate, dizziness; it needs 5-6 months before the user notices the desired results.

Provigro http://www.provigro.com	Horny goat weed; maca root; saw palmetto; Tongkat Ali; *Mucuna pruriens* 10% L-dopa; Muira puama; L-arginine; *Panax ginseng*.	34.97 $ per box; three-month supply for 57.80 $	Online suppliers (orders can be placed by fax, phone, and mail).	Stated as “natural vitamins, minerals and herbs”; customers testimonials; money back guarantee; it does not contain Yohimbe.	Reported nausea, dizziness, and vomiting; it may take up to six months to work.

Quantum Pills http://www.quantumpills.com	L-arginine; L-lysine HCL; maca root; Muira puama; Swedish flower pollen extract; horny goat weed; Catuaba bark; *Gingko biloba*; zinc oxide.	44.95 $ per box; six-month supply for 189.95 $	Online suppliers (orders can be placed by fax, phone, and mail too).	Stated as a “climax enhancer,” assures increase in ejaculation volume; natural; money back guarantee; customers testimonials.	No permanent results; no scientific proofs and no adequate ingredients explanation.

Sinrex http://www.sinrex.com	Bioperine; copper chelate; creatine; Cuscuta seed; *Gingko biloba*; green tea extract; Hawthorn berries; Epunedum Sagitum; inosine; L-arginine; maca; omega 3; saw palmetto; Siberian ginseng; soy proteins; *Tribulus terrestris*; vitamin E.	39.95 $ per box; six-month supply for 199.95 $	Online suppliers (orders can be placed by fax, phone, and mail too); Ebay.	Presented as “all natural; money back guarantee; customers testimonials; semen volume and orgasm intensifier.”	At least one month for initial results; possible allergies to ingredients.

Sizepro http://www.sizepro.com	Vitamin E; vitamin B3; horny goat weed; hawthorn berry; damiana; Muira puama; *Gingko biloba*; Chinese ginseng; *Tribulus* fruit extract; Catuaba bark; saw palmetto; inosine; L-arginine; oat straw; cayenne fruit; soy protein.	Two-month supply for 99.95 $; six-month supply for 245.95 $	Online suppliers (orders can be placed by fax, phone, and mail too); Ebay.	Natural; “Lasting increase of penis thickness and size; money back guarantee; customers testimonials.”	

Vazomyne http://www.vazomyne.net	Not completely listed. Vazomyne includes L-arginine, saw palmetto, *Gingko biloba*, damiana, Polygonum multiflorum, nettle leaf, Muira puama.	39.99 $ per box; three-month supply for 99.98 $	Online suppliers (orders can be placed by fax, phone, and mail too); Ebay.; Amazon (currently not available).	Presented as: “natural; money back guarantee; customers testimonials.”	Ingredients are not fully listed nor explained in details; “It should not be taken in case of heart or thyroid problems; will not increase penis size.”

**Table 2:** Subjective reports of forums' users about sexual enhancers side effects.

Gingko biloba	(i) Head rush (ii) Anxiety (iii) Feeling of blood flowing through skin/feeling blood warming (iv) Tinnitus http://www.drugs-forum.com/forum/showthread.php?t=77902 (i) Increased heart rate (ii) Dizziness (iii) Chest pain (iv) Phosphenes http://forum.lef.org/default.aspx?f=35&m=16405 Anxiety and nausea (Ginkgo tea consumption while assuming clomipramine) http://www.socialanxietysupport.com/forum/f11/ginkgo-biloba-197737/

Maca	(i) Altered menstrual cycle (length modifications, anovulatory cycles) (ii) Painful intestinal cramps (iii) Severe gastritis (iv) Increased blood pressure (v) Moodiness http://archive.tcoyf.com/forums/t/119232.aspx (i) Increased heart rate (ii) Insomnia (iii) Depression/anxiety (iv) Worse premenstrual syndrome symptoms (v) Acne breakout http://www.highonhealth.org/what-nobody-tells-you-about-maca-root-powder-dangers-and-side-effects/ Mood swings http://babyandbump.momtastic.com/pregnancy-tests/419834-gallery-o-tests-1075.html#post9282851

Horny goat weed	(i) Fever (ii) Increased heart rate (iii) Aggressiveness (iv) Irritability http://www.muscleandstrength.com/supplements/ingredients/horny-goat-weed.html#5
